# Investigation of Neural Microenvironment in Prostate Cancer in Context of Neural Density, Perineural Invasion, and Neuroendocrine Profile of Tumors

**DOI:** 10.3389/fonc.2021.710899

**Published:** 2021-07-01

**Authors:** Dawid Sigorski, Jacek Gulczyński, Aleksandra Sejda, Wojciech Rogowski, Ewa Iżycka-Świeszewska

**Affiliations:** ^1^ Department of Oncology, Collegium Medicum, University of Warmia and Mazury, Olsztyn, Poland; ^2^ Department of Oncology and Immuno-Oncology, Warmian-Masurian Cancer Center of the Ministry of the Interior and Administration Hospital, Olsztyn, Poland; ^3^ Department of Pathology and Neuropathology, Medical University of Gdańsk, Gdańsk, Poland; ^4^ Department of Pathomorphology, Copernicus Hospital, Gdańsk, Poland; ^5^ Department of Pathomorphology, Collegium Medicum, University of Warmia and Mazury, Olsztyn, Poland; ^6^ Department of Health, Pomeranian University in Słupsk, Słupsk, Poland; ^7^ Department of Oncology, Chemotherapy, Clinical trials, Regional Hospital, Słupsk, Poland

**Keywords:** neural microenvironment, prostate cancer, NPY, nerve density, perineural invasion

## Abstract

**Background:**

Cancer stroma contains the neural compartment with specific components and action. Neural microenvironment processing includes among others axonogenesis, perineural invasion (PNI), neurosignaling, and tumor cell neural/neuroendocrine differentiation. Growing data suggest that tumor-neural crosstalk plays an important function in prostate cancer (PCa) biology. However, the mechanisms involved in PNI and axonogenesis, as well as their patho-clinical correlations in this tumor are unclear.

**Methods:**

The present study was carried out on FFPE samples of 73 PCa and 15 benign prostate (BP) cases. Immunohistochemistry with neural markers PGP9.5, TH, and NFP was performed on constructed TMAs and selected tissue sections. The analyzed parameters of tumor innervation included small nerve density (ND) measured on pan-neural marker (PGP9.5) and TH s4tained slides, as well assessment of PNI presence and morphology. The qualitative and topographic aspects were studied. In addition, the expression of neuroendocrine marker chromogranin and NPY was assessed with dedicated indexes. The correlations of the above parameters with basic patho-clinical data such as patients’ age, tumor stage, grade, angioinvasion, and ERG status were examined.

**Results:**

The study showed that innervation parameters differed between cancer and BP. The neural network in PCa revealed heterogeneity, and ND PGP9.5 in tumor was significantly lower than in its periphery. The density of sympathetic TH-positive fibers and its proportion to all fibers was lower in cancer than in the periphery and BP samples. Perineural invasion was confirmed in 76% of cases, usually multifocally, occurring more commonly in tumors with a higher grade. NPY expression in PCa cells was common with its intensity often rising towards PNI. ERG+ tumors showed higher ND, more frequent PNI, and a higher stage. Moreover, chromogranin-positive cells were more pronounced in PCa with higher NPY expression.

**Conclusions:**

The analysis showed an irregular axonal network in prostate cancer with higher neural density (panneural and adrenergic) in the surroundings and the invasive front. ND and PNI interrelated with NPY expression, neuroendocrine differentiation, and ERG status. The above findings support new evidence for the presence of autocrine and paracrine interactions in prostate cancer neural microenvironment.

## Introduction

There is growing preclinical and clinical data confirming that the peripheral and central nervous system contributes to the initiation and progression of cancer (1, 2). The neuronal influence is provided by sympathetic and parasympathetic activity through electrochemical synaptic impulses, paracrine and systemic signaling. Connections between cancer and its neural microenvironment constitute a new concept in cancer pathobiology and begin the era of cancer neuroscience (3–6).

The correlations between prostate cancer (PCa) and the nervous system are prominent. PCa often develops multifocally within the peripheral region of the organ which is the most innervated. A lower incidence of PCa was noted in patients with spinal injury. Moreover, experimental studies showed that sympathectomy decreased the risk of cancer (7, 8). The phenomenon of cancer axonogenesis and neurogenesis in PCa was first described by Ayala et al. (9). The rich innervation of the prostate is clinically important for oncologists and urologists, especially in the context of the surgical technique and its consequences for the patient’s quality of life (10). Perineural invasion (PNI) is a well-known phenomenon in cancer, being explained for decades as a passive type of neoplastic infiltration. The incidence of PNI in PCa is the highest among urogenital malignancies. It is one of the ways of cancer spreading. Additionally, it is postulated as an additional factor of the development of bone metastases (11, 12). The active role of the neuronal network within the tumor and its surroundings is another new focus of investigations in cancer neuroscience. Since little is known about the biological mechanisms contributing to PNI, *in vitro* and *in vivo* studies showed that neurotrophic factors and axon guidance molecules were involved in this process (12–16). Neurotransmitters, neurotrophins and neuropeptides play a crucial role in the neural regulation of cancer cells, but their exact mechanisms remain undefined (12, 16). Furthermore, different hypotheses were presented to explain the origin of nerve fibers in the cancer microenvironment. One of them is axonogenesis defined as an increase observed in case of axon extensions and their quantity or growth in the nerves within the tumor (9). Several parameters were implemented to examine tumor innervation, e.g. axonal or neural density (ND), neural area, neural score and neural index (2, 17–21). ND seems to be a simple method, but available research on different types of cancer provided divergent results due to differences in definitions, methodology, and the type of analyzed samples (17, 20–23). Since the chemical, genetic or surgical modifications of nerves influence tumor development, the relations cancer – the nervous system became a new concept of treatment (2).

The neural stromal compartment is responsible for a specific microenvironment which is different in benign and malignant prostatic glands (5). The glandular part of the normal prostate is supplied by cholinergic fibers while the stromal part – by adrenergic ones and peptidergic nerve fibers. Neuroendocrine cells (NE) which are dispersed within the prostate epithelium release several neurotransmitters, which play an integrational role. This innervation and interaction model is aberrant in cancer (2). Preclinical studies supported evidence confirming that the sympathetic nervous system was involved in the early and local phases of PCa growth, and the parasympathetic system had a role in its dissemination (2). The neurotransmitters, neurotrophic factors and axon guidance molecules which are released by nerves and cancer cells drive the tumorigenesis of PCa. In addition, prostate and PCa cells are characterized by the altered expression of genes which are involved in neural regulation.

The E26 transformation-specific (ETS) transcription factors are a family of transcription factors which control numerous biological processes like the apoptosis, angiogenesis, growth, proliferation, migration and differentiation of cells. They are also involved in the pathogenesis and progression of cancer (24). The *TMPRSS2-ERG* fusion is an early event in PCa which most frequently occurs between exon 1 of the TMPRSS2 gene and exon 4 of the ERG gene which is an ETS member. The *TMPRSS2-ERG* fusion is one of the most common genetic alterations in PCa, leading to the androgen-regulated overexpression of ERG oncogene, which affects the expression of numerous signaling pathways and genes including Neuropeptide Y (NPY) (25–27).

NPY is the most abundant peptide in the central nervous system. However, it is also released by nerves and can be synthetized by tumor cells (28). The most important pathway involved in prostate cancer biology is the androgen pathway, but nervous signaling emerges as the second one (29). Interestingly, the amount of NE cells increases during antiandrogen PCa therapy, leading to the so called “neuroendocrine switch” and therapy resistance (castration-resistant prostate cancer) (30).

The study was performed to analyze the morphological and quantitative aspects of axonogenesis and neuroinvasion in prostate cancer and their correlations with the neuroendocrine features and basic patho-clinical characteristics of tumors.

## Material and Methods

The research was carried out according to The Code of Ethics of the World Medical Association (Declaration of Helsinki) and approved by MUG (Medical University of Gdańsk) Bioethics Committee (NKBBN/448/2015). The examined group was based on 97 formalin-fixed paraffin-embedded archival samples of prostate carcinoma (PCa) obtained from patients who underwent radical prostatectomy as the first-line treatment at two oncology centers between 2013 and 2016. Before prostatectomy, patients had not been treated with hormone therapy and/or radiotherapy. The tumors were examined according to WHO 2016 Classification of Tumours of the Urinary System and Male Genital Organs, TNM 8th Edition, and the recommendations of the Polish Society of Pathologists (31–33). The patho-clinical data encompassed: patients’ age, tumor stage, histological Grade Group (GG), the presence of perineural invasion, and angioinvasion. The characteristics of the group are collated in **Table 1**. The control group consisted of 15 benign prostate (BP) cases operated due to benign prostate hyperplasia (12) or bladder cancer (T2N0) (3). Hematoxylin and eosin (H&E) slides from all cases were reviewed by two observers. The most representative fields of neoplastic tissue and, if present, areas with PNI were selected. 24 cases were excluded, because of insufficient material for further analysis.

**Table 1:** The patho-clinical characteristics of the study group.

Age of patients (55-80 years)	N (%)
<65 years old	25 (34.25)
≥ 65 years old	48 (65.75)
***pT stage***	**N (%)**
2a	2 (2.74)
2b	2 (2.74)
2c	27 (36.98)
3a	22 (30.14)
3b	20 (27.40)
***pN stage***	**N (%)**
0	67 (91.78)
1	6 (8.22)
***Grade Group***	**N (%)**
1	12 (16.43)
2	33 (45.21)
3	13 (17.81)
4	7 (9.59)
5	8 (10.96)
***Perineural Invasion (PNI)***	**N (%)**
No	17 (23.29)
Yes	56 (76.71)
***Extraprostatic extension***	**N (%)**
No	31 (42.47)
Yes	42 (57.53)
***Angioinvasion or/and Lymphatic invasion***	**N (%)**
No	57 (78.08)
Yes	16 (21.92)
***ERG***	**N (%)**
Negative (0)	38 (52.05)
Positive (1,2)	35 (47.95)

The study was performed with tissue microarray technique (TMA) with Tissue-Tek Quick-Ray Tissue Microarray System (Sakura, Japan). Seven TMAs were constructed with tissue cores, 5 mm in diameter, collected from 73 cases of the PCa group and 15 from the control BP group. Each case was represented by 2-3 cores, parallel to the heterogeneity of specimens and a separate sample from PNI fields. In total, we examined 156 prostate cancer and 14 benign prostate cores for each immunostaining. Additionally, whole section slides were used in six cancer cases for topographical and morphological analysis. The blocks were cut serially into 4 µm-thick sections. The next step comprised immunohistochemistry with the use of: monoclonal anti-protein Gene Product 9.5 antibody (PGP) (anti-PGP 9.5, ab 8189, mouse, Abcam, 1:1000), polyclonal anti-tyrosine hydroxylase antibody (anti-TH, ab 112, rabbit, Abcam, 1:500), monoclonal anti-neurofilament proteins (anti-NFP, Dako Ready-to-use, mouse, Dako), polyclonal anti-neuropeptide Y (anti-NPY, ab 30914, rabbit, Abcam, 1:1000), anti-chromogranin A (mouse, Ready-to-use, Thermo Fisher Scientific), and monoclonal anti-ERG antibody (ab 136152, mouse, Ready-to-use, Abcam). PGP and NFP were used as pan-neuronal markers while TH was used as a sympathetic marker. The immunohistochemistry was carried out with appropriate positive and negative controls, with the routine performed according to the producer guidelines and dedicated protocols established in our laboratory. Histopathological assessment and analyses were carried out with a light microscope (Olympus BX51). All slides were scanned with Hamamatsu C12740 scanner.

Immunohistochemistry with PGP, TH and NFP was performed for the morphological and quantitative analysis of neural structures (nerves, nerve fibers/axons). The number of axons and nerves was counted manually in five fields in the hot spot tumor area, and at least two fields in the tumor periphery (up to 1 mm outside of the tumor infiltration) in hot spot areas. Hot spot areas were identified under 200x magnification. Nerve density was defined as the average of structures per one field under 400x magnification (the area of 0.15 mm^2^). The diameter of counted neural structures was below 20 µm. Any immunopositive single separate dark brown structure taking the shape of a dot, line, or linear structure was considered and counted.

Perineural invasion was defined as cancer cell presence in any of the nerve layers in the form of an invasion, surrounding, or passing through a nerve.

NPY expression was performed semi-quantitatively as the expression score (ES). The paravertebral ganglion neurons were used as the positive control. ES (0–2) was based on the intensity and rate of staining. The intensity of staining was calculated on a 0-3 scale (0 – negativity, positivity: 1 – weak, 2 – moderate, 3 – strong), in two selected tissue regions with the strongest staining. The field was considered positive if at least 5% of cells showed positivity. ES was calculated with the following formula: 1x[% of cells staining weakly (0-1)] +2x[% of cells staining moderately (2)] +3x[% of cells staining strongly (3)]. Finally, ES was calculated according to the modified scale by Pirker et al.: ES0 (0-99), ES1 (100-199), and ES2 (200-300) (34). A 0-2 scale was used to assess the expression of NPY in PNI areas in comparison to tumor infiltration. The strength of staining was classified as: the same/weaker (0), stronger (1), or much stronger than 1 (2).

Chromogranin expression was assessed as a measure of neuroendocrine differentiation. The average of positive cells was counted in one field (surface area – 0.15 mm^2^, magnification: 400x) in 5 hot spot areas. In addition, we assessed the level of chromogranin expression according to Ishida et al. (35). It was classified as high (H; ≥10 positive cells/1 field) or low (L; <10 positive cells/1 field). ERG expression was assessed in the nuclei of cancer cells on the scale: 0 (negativity), 1 (weak, light brown staining), and 2 (strong, dark brown, or at least 50% of dark nuclei). The endothelial cells were used as the positive control. Only two categories: negative (0) and positive (1 and 2) were used for further statistical analysis.

Statistical analysis was performed with MedCalc software. The differences between groups were tested using the Mann-Whitney U test and the t-student test. Differences at p<0.05 were considered statistically significant.

## Results

### The Morphological Analysis of Innervation

**Figure 1:**
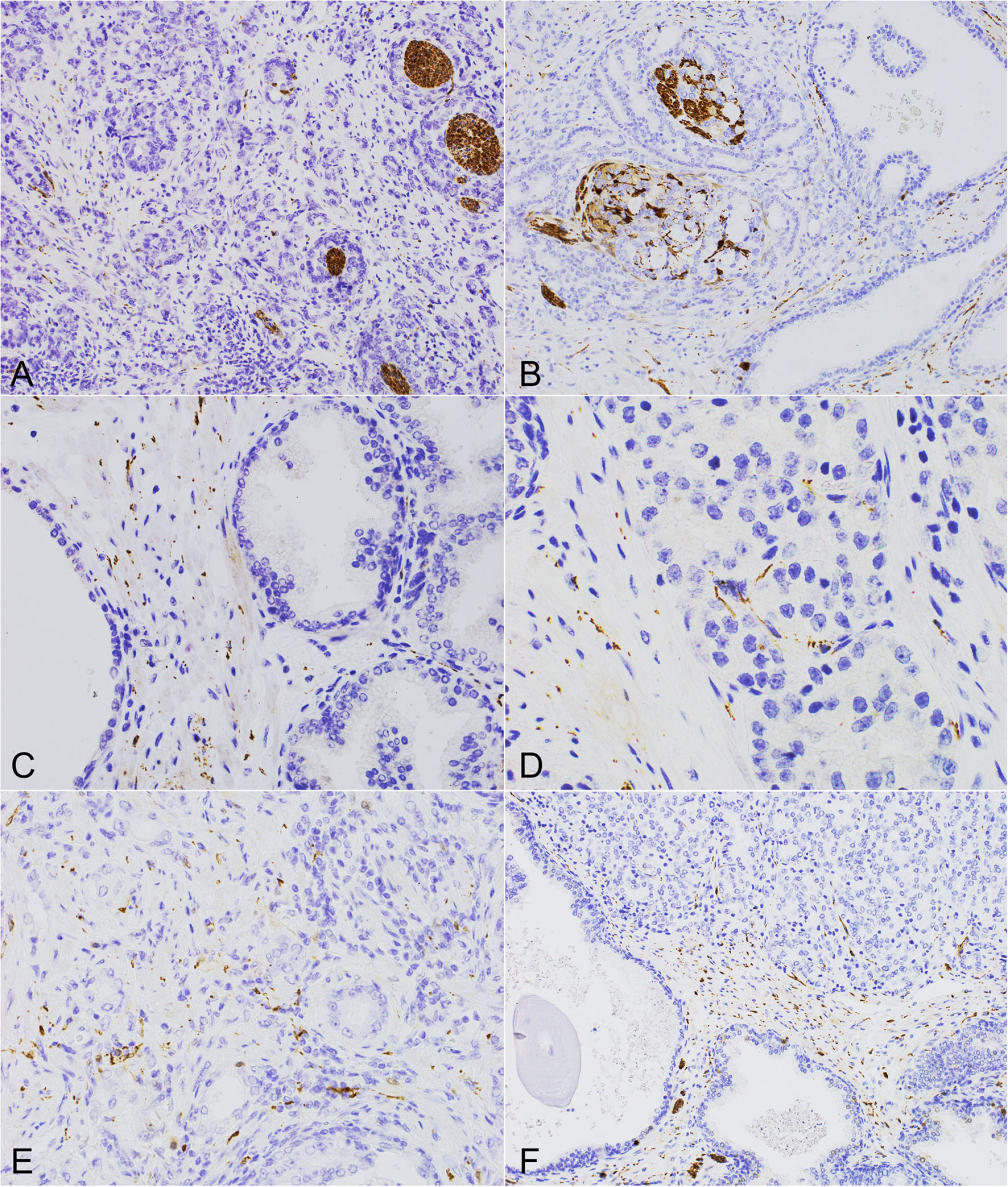
The morphology of perineural invasion and axonogenesis in PCa and the benign prostate. **(A, B)** Different forms of perineural invasion and small nerves within the cancerous infiltration (PGP 9.5, 200x). **(C)** Multiple axons and small nerves within the stroma of the benign prostate (PGP 9.5, 400x). **(D, E)** Axons within the neoplastic infiltrate located in the stroma and surrounding cancer cells (**D**, TH, 600x), (**E**, TH, 400x). **(F)** High neural density in the surrounding benign prostate with axons growing into the cancer tissue of high Gleason score PCa (PGP 9.5, 200x).

**Figure 2:**
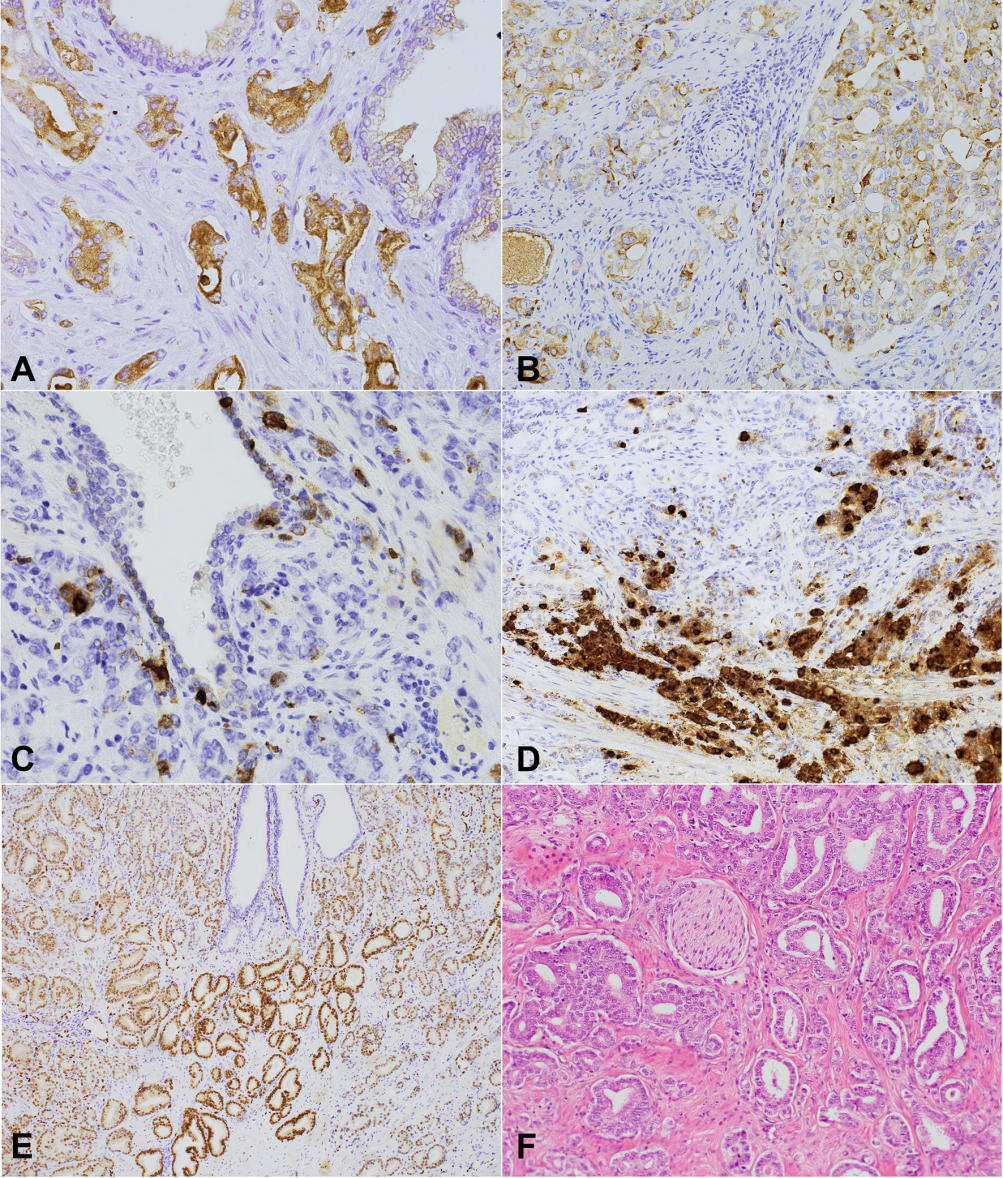
NPY, chromogranin, and (NPY, 400x) ERG expression and perineural invasion in prostate cancer. **(A, B)** Cytoplasmic-membranous NPY expression in neoplastic glands. **(A)** Strong expression score (2). **(B)** 200x Heterogenous intermediate NPY expression score and membranous apical immunostaining within the preexistent prostate glands (NPY, 200x). **(C)** Chromogranin – a strongly positive scattered single cell pattern (chromogranin, 400x). **(D)** A high level of neuroendocrine differentiation within PCa (chromogranin). **(E)** Nuclear ERG expression with diverse intensity within the cancer cells, positive endothelial cells, and immunonegative benign prostatic glands (ERG, 100x). **(F)** A histological section of prostate cancer with focal perineural invasion and other invisible neural components of the microenvironment (HE, 200x).

The arrangement of nerve structures differed between PCa and BP (**Figures 1A–F**, **2F**). PGP was the most sensitive antibody with the best detection contrary to NFP which was the least sensitive and showed the lowest number of structures. In BP the highest concentration of nerve structures was identified in the periphery of the organ, where the diameter of nerve fibers was the highest and diminished towards the center of the prostate. Numerous axons in the fibromuscular stroma and axons surrounding vessels were revealed at the depth of 3-5 mm. Axons were distributed quite regularly in BP, with irregularity found in nodular hyperplasia. Small nerve fibers also surrounded the blood vessels. As regards cancer, the nerve fibers were distributed irregularly. Axons penetrated between the neoplastic glands and inflammatory infiltrate in the stroma and entwined them in PCa PGP+. Axons that closely adhered to separate glandular cells created a basket or net-like structures. TH-positive axons occurred mainly in the stroma of the prostate contrary to neoplastic interglandular infiltration. The highest concentration and diameter of nerve structures were present in the peripheral region of neoplastic prostate infiltration. Moreover, differences occurred between low (GG 1,2,3) and high-grade (GG 4,5) tumors. In low-grade tumors, the nerves were dispersed within the infiltration among the preexistent benign glands as opposed to high-grade cases creating a more solid mass, where the neuronal network dominated in the invasive tumor front.

PNI included the infiltration of larger nerves, ganglia and nerve structures, most commonly in the form of segmental or circular invasion, and in 90% of cases they occurred multifocally. The disintegration (combing out) of the nerves by neoplastic cells was also observed, usually located more centrally in the tumor.

### Neural Density

The assessment of ND with TH sympathetic marker showed that TH ND was lower in PCa than in BP (8.40 *vs.* 15.6 structures; p<0.01). No difference was observed between PGP ND in PCa and BP (31.45 *vs.* 38.15 structures; p=0.21). ND was higher in cancer periphery than in PCa (PGP – 42 *vs.* 31.45 structures; p=0.01; TH – 21.5 *vs.* 15.6; p<0.01). There was no difference between the periphery of cancer and BP in both axonal markers, i.e. PGP (p=0.52) and TH (p=0.57). In addition, we analyzed the statistical correlation between PGP and TH ND in PCa (r=0.44; 95% CI 0.23-0.6; p<0.01). The comparison of PGP/TH ND ratio in PCa and the periphery was statistically significant (PGP; r=0.35; 95% CI 0.13-0.54; p<0.01; TH; r=0.36; 95% CI 0.14-0.55; p<0.01). Furthermore, the PGP/TH ratio was higher in PCa than in the periphery (p<0.01) and BP (p=0.02).

### The Correlations Between ND and PNI, and the Clinico-Pathological Features of Tumors

No correlations were found between tumor differentiation grade (Grade Group <3 *vs.* ≥3), ND in PCa (PGP p=0.86; TH p=0.77), and the peripheral area of PCa (PGP p=0.66; TH p=0.52). The only subgroup of Grade Group 1 (15.90, 95% CI 4.37-19.89) tumors were characterized by higher TH ND than Group 2 (8.00, 95% CI 5.13-9.26, p=0.04). Moreover, the difference between tumor stages (pT2 and pT3) was not statistically significant (**Table 2**).

**Table 2:** ND and PNI in the study group according to Grade Group, pT stage and ERG status.

	PGP ND		TH ND		PNI	
**PCa**	**(structures/field of view)**	p-value	**(structures/field of view)**	p-value	N (%)	p-value
**Range:**	1.5-87.4		1-27.4			
**Average:**	31.1		9.9			
**Median:**	31.45 (95% CI 24.3-36)		8.40 (95% CI 6.97-10.1)			
**pT**	Median					
**pT2**	26.50 (95% CI 1.5-73.9)	p=0.28	8.00 (95% CI 1-27.4)	p=0.96	37 (66)	p=0.02
**pT3**	34.05 (95% CI 3.4-87.4)		8.40 (95% CI 1-26.4)		19 (34)	
**GG**						
**1**	33.50 (95% CI 10.34-45.47)	p=0.04*	15.90 (95% CI 4.37-19.89)		5 (41.66)	p<0.01**
**2**	28.60 (95% CI 21.02-36.05)		8.00 (95% CI 5.13-9.26)		27 (81.82)	
**3**	37.30 (95% CI 11.10-53.97)		12.90 (95% CI 3.74-18.79)		10 (76.92)	
**4**	35.10 (95% CI 19.40-50.18)		10.20 (95% CI 4.19-22.40)		7 (100)	
**5**	24.17 (95% CI 17.28-41.64)		7.13 (95% CI 5.57-10.56)		7 (87.50)	
**ERG**						
Positive***	36.00 (95% CI 29.38-42.62)	p=0.03	8.80		PNI + PNI -	p=0.03
**Negative**	26.58 (95% CI 21.10-32.05)		8.00		31 (42.46) 4 (5.48)	
					25 (34.25) 13 (17.81)	
**Periphery**		p-value		p-value		
**Range:**	5-143		1-100			
**Average:**	46.2		26.89			
**Median:**	42 (95% CI 33.8-55)		21.5 (95% CI 17.9-29.5)			
**pT**						
**pT2**	42.50 (95% CI 5-124)	p=0.85	25.00 (95% CI 1-100)	p=0.49		
**pT3**	40.00 (95% CI 7-143)		20.75 (95% CI 1-66)			
**BP**						
**Range:**	10.2-114.9		3.5-185			
**Average:**	36.15		32.7			
**Median:**	38.15 (95% CI 15.2-61.2)		15.60 (95% CI 10-40.79)			

GG, Grade Group; PNI, perineural invasion; * for GG1 *vs.* GG2, ** for GG1 *vs.* GG2-5,*** the mean.

PNI was observed more commonly in Grade Group >1 tumors than GG 1 (OR 7.13, 95% CI: 1.88-27.08; p<0.01). PNI occurred more frequently in pT3 tumors than in pT2 (OR=4.67; 95% CI 1.43-15.22; p=0.02). Additionally, we did not observe the dependence of perineural invasion on the invasion of blood and lymph vessels (p=0.09), which was more common in pT3 tumors (p=0.01).

### The Analysis of NPY and Chromogranin Expression

Benign prostate and PCa cells showed NPY expression (**Figures 2A, B**). As regards BP, only luminal cells were characterized by membrane apical expression and, rarely, weak cytoplasmic staining, with the negativity of basal cells (**Figure 2B**). Cancer glands were characterized by both cytoplasmic and membrane staining with different intensity. Neuroendocrine cells in BP and PCa were NPY-positive, but the strongest staining was observed in case of autonomic nerve ganglia (**Figures 2C, D**). Other compounds of the tumor stroma were negative. In 65.75% (45 cases) the staining was non-homogeneous. The results of immunostaining are presented in **Table 3**. We checked if the parameters of cancer innervation depended on NPY expression. The average of ND in PCa and the periphery was the same (**Table 3**). However, a tendency towards lower ND was noted in the peripheral tumor area of PCa in tumors with a high NPY expression (p=0.07). In some cases NPY expression was higher in the peripheral area or in the proximity of the nerves. A high NPY expression (NPY2) in PNI area occurred more commonly in NPY-positive than NPY-negative cases (OR: 5, 95% CI: 1.14-24.64; p=0.03). No correlation occurred between NPY and tumor stage (p=0.74), EPE (p=0.23), Grade Group (p=0.95), and ERG (p=0.60).

**Table 3:** The summary of chromogranin and NPY expression results.

Marker	N (%)
***Chromogranin staining***	
1. Single-cell pattern	57 (78.08)
Cluster pattern	15 (20.55)
Diffuse pattern	1 (1.37)
2. High expression	57 (78.08)
Low expression	16 (21.92)
***NPY***	
Expression score	
0	4 (5.48)
1	25 (34.25)
2	44 (60.27)
Expression in PNI	
Lower/equal	27 (73.68)
Higher	10 (26.32)

GG, grade group; NPY, neuropeptide Y; PNI, perineural invasion.

In BP, chromogranin positivity was present in neuroendocrine cells located between luminal cells. In cancer cells, three types of expression patterns were distinguished – a single-cell, cluster, and diffuse pattern (**Table 3**) (35). Chromogranin immunoreactivity was not different in PNI regions compared to the rest of the infiltration. The average number of chromogranin-positive cells varied between 1 to 38/field in PCa (median 1.65, 95% CI: 1-2.38), while in BP – between 0 to 15.8 (median 6.40, 95% CI: 4.65-7.52). The median of positive cell count was lower in PCa than in BP (p<0.01, 95% CI: 1.60-5.40). The level of chromogranin expression (High/Low) did not differ significantly between ND in the tumor (PGP 9.5, p=0.78; TH, p=0.85) and the periphery (PGP 9.5 p=0.37; TH p=0.78), PNI (p=0.67), pT stage (p=0.12), Grade Group (p=0.19), vascular invasion (p=0.11), EPE (p=0.12), age (p=0.12), and ERG (p=0.37). Interestingly, a high chromogranin level (H) was observed in tumors with a high NPY expression (NPY2, OR=3.84; 95% CI: 1.10-13.35, p=0.03).

### The Analysis of ERG Expression

A total of 47.95% of studied PCa cases were ERG-positive (**Figure 2E** and **Table 1**). The analysis of ND in correlation with ERG status revealed that the mean of ND PGP 9.5 in the ERG+ group was higher than in ERG- group (CI 95% 1.03-17.81; p=0.03), but ND TH was similar in PCa (p=0.89). PNI was present four times more commonly in ERG-positive tumors than in ERG-negative ones (OR=4.03; 95% CI 1.17-13.90, p=0.03). ERG positivity was associated with EPE (OR=3.22; 95% CI:1.19-8.63, p=0.02), but it was not correlated with Grade Group (p=0.10), age (p=0.62), or angio- and lymphatic invasion (p=0.14).

## Discussion

Complex interactions between nerves and PCa cells were first described by Ayala et al. who showed that the co-culture of PCa cell line with dorsal root ganglion stimulated the outgrowth of neurites and the migration of tumor cells toward the neural structures (6). PCa represents a tumor with a specific microenvironment, where reciprocal interactions between nerves and other cellular and extracellular components were observed molecularly and clinically *in vitro* and *in vivo* (5, 7, 36, 37). Neurotransmitters released within the tumor microenvironment modulate the expression of genes by targeting tumor receptors affecting the biological potential of cancer. PCa cells are prone to neurotransmitter expression, partially due to the special signature of genes known as the “brain profile’’ particularly observed during the CRPC stage of the disease (29, 38). The systemic and regional activity of the autonomic nervous system seems to differ between solid tumor types. In some of them tumor growth is promoted by adrenergic transmission. Conversely, cholinergic ones have stimulating or inhibitory properties or function depending on the type and stage of cancer (39, 40). Neural stimulation was found to modify immune response, angiogenesis, apoptosis, cancer cell metabolism, and invasiveness (3, 16, 39, 41). Neurogenesis and axonogenesis may be considered as new hallmarks of cancer, similarly to the neuro-glial activation or angiogenesis (42, 43). The available results underlay the hypothesis of the importance of neural network and altered nerve density in PCa (2). An experimental clinical study by Magnon et al. demonstrated that ND was higher in the PCa area and cancer-surrounding zone in the high-risk group of patients. Moreover, this group showed a different sympathetic and parasympathetic regulation of tumor growth which might be modified genetically, surgically or pharmacologically (2).

Our study was conducted to examine axonogenesis and some aspects of the crosstalk between nerves and neoplastic cells in PCa. Our analysis revealed the heterogeneous distribution and topography of nerves in cancer compared to benign prostate where the distribution of innervation was regular and adjusted to histological structures. Morphological analysis showed the presence of small nerve fibers in the invasive tumor front, which also implies the nervous promotion of tumor invasive growth. Moreover, PNI presented more distinct morphological features in the peripheral areas of the prostate and extraprostatic extension zones than in the deeper part of the prostate. More centrally, cancer infiltrate was able to disintegrate the nerves, probably activating the axonal sprouting. The distribution of nerves within the tumor infiltrate microenvironment was not homogeneous. The quantity of the analyzed structures depended on the amount of stroma. High-grade compact and cellular tumors (GG 4-5) did not contain many internal axonal structures contrary to low-grade tumors with more dispersed growth among the preexistent structures. The presence of neural structures in PCa stroma is diverse and may be a result of the recruitment of loco-regional nerves by the tumor, but also of axonogenesis or neurogenesis (1, 2, 44). It was proposed that the origin of new neurons in the tumor microenvironment might be peripheral or even central (1, 9). The concept of central neurogenesis was proposed by Mauffrey et al. who found the increased number of cells with doublecortin in the murine model of PCa and discovered that those cells originated from the cerebral subventricular zone, settled in the prostate and differentiated into adrenergic nerve fibers (1). The peripheral hypothesis of neurogenesis is based on neuronal or stem cell differentiation processes. Reeves et al. showed that the most common type of nerves was the mixed type and pure parasympathetic innervation (45). Under special conditions, PCa cell line may trans-differentiate into neuronal phenotype cells (29, 46). Moreover, the transcription gene-based analysis of prostate basal cells showed the expression of genes that were involved in neural and neuronal development (29).

ND is one of the most commonly used quantitative parameters of tumor innervation (9, 17, 19, 47–49). It was analyzed with digital image systems of slides immunolabeled with different markers, such as TH, VACHT, PGP 9.5, NF-H, NF-L antibodies, and special image processing algorithms. Moreover, radiological methods may also be used, e.g. superparamagnetic iron oxide peptide nanoparticles in magnetic particle imaging (49). All of the techniques have various specificity and sensitivity levels. Non-compatible tumor populations make the comparison of ND between studies difficult in the context of clinical and tissue material, and methodology. Several authors defined ND as the total nerve area, the number of nerves per unit area, or different ND scores (17, 20, 21, 23, 48). The completely simplified method included only a statement of the presence or absence of nerves and the level of neural marker staining intensity (22, 50).

We performed a qualitative and quantitative analysis of innervation on TMAs and a set of whole tissue sections to optimize and validate the results. We adopted the technique of the manual calculation of structural elements on digital images (PGP 9.5, NPY, chromogranin, ERG) and light microscope images (TH). PGP 9.5 appeared to be the most sensitive pan-neural marker. Similarly to some other reports the direct analysis of parasympathetic nerves was not performed in our study, due to the lack of a reliable marker in formalin-fixed, paraffin-embedded samples at the time of measurements (45). Our technique of ND assessment was laborious, but we precisely counted low diameter axons, even as small as <4 µm. Such a technique has not been presented in the literature yet. Counting individual axons provides more precise information about the neoplastic microenvironment in PCa than counting bigger nerve branches. The results of ND differ between cancer types and may be increased or decreased, in addition to its different clinical relevance. To date, ND has been analyzed in prostate, breast, colon, liver, pancreas, and thyroid cancers (17, 20, 22, 45, 48, 50). Neural density reveals diverse relations with histopathological criteria, tumor stage and clinical relevance in different tumors. For example, ND was found increased in hepatocellular carcinoma, thyroid and breast cancer and in some of them correlated with a higher metastatic potential, shorter disease-free survival and the overall survival. The degree of axonogenesis may be a more important prognostic factor than lymph node involvement in colorectal cancer (17, 22, 49). We showed that the absolute amount of PGP-positive structures was lower in the cancer bed, in parallel to significantly lower TH ND median in cancer compared to the benign prostate. The density of sympathetic fibers and their proportion to all stained fibers were lower in cancer than in the benign prostate (p<0.0001). Conversely, PGP ND was significantly higher in the proximal periphery, close to cancer infiltration than in the cancer area, which suggests that nerves may drive tumor progression and invasion. Our results are consistent with the Japanese study of Iwasaki et al. in pancreatic cancer, where intrapancreatic neural density and the number of nerves were lower and decreased towards the center of the tumor (20). Importantly, low neural density and high perineural invasion were associated with shorter overall survival in pancreatic cancer patients (20). In several PCa studies, high nerve density was associated with poorer clinical outcomes and a higher tumor proliferative index, which could be explained by the correlation between ND and prosurvival pathways, hormonal receptor status, co-regulators and co-repressors (2, 18). The increased nerve density in PCa was shown by Magnon et al. only in high-risk PCa patients. They assessed ND by calculating nerve fibers in the representative normal prostate and PCa areas with tumor grade distinction using digital microscopy software (2). This study was based on 43 PCa cases divided into low- and high-risk-defined PSA >10 ng/ml, Gleason Score ≥7, and ≥pT2b. Their division of the PCa group was different than the widely accepted clinical risk-group guidelines, since PSA level >20 ng/ml defines tumors as high-risk independently from Gleason score or T stage (51, 52). In the study by Magnon et al. only 6 tumors had PSA above 20 ng/ml. Moreover, no pT2b tumors were present in this high-risk group. However, T3a-T4a cases were described, which means that according to PSA level or Gleason score they were classified as a high-risk group. Other authors demonstrated that the number of nerves might be tumor stage-dependent, although correlations between tumor grades were less pronounced (45). Reeves et al. found no dependencies between PNI status in different nerve subtypes and Gleason score contrary to the quantity of nerves in PNI tumors which was twice higher (45). Our study showed that the difference between tumor stage (pT2 and pT3) and ND was not statistically significant. Additionally, we demonstrated that regarding grade groups, only Grade Group 1 tumors had lower TH ND than GG2. However, our group was not well balanced. GG2 was the most common (45%). There was a relatively small representation of GG 4 and 5, which is typical of studies on prostatectomy series (53, 54). The associations between ND *vs.* GG or GS need evaluation on larger and more representative groups of PCa cases.

The incidence of PNI in various PCa series varied between 12.4 and 83%, but it differed between biopsy and prostatectomy specimens (21, 23). Our group included 76.71% of cases with PNI. The prognostic significance of PNI is still not well established in PCa (55–57). We analyzed PNI according to the classic definition: the presence of cancer cells in or within nerves, the circumference or divisions. PNI is defined as a simple adhesion to nerve trunks or splitting nerve structures. As regards our group, PNI occurred more frequently in pT3 tumors than in pT2 and Grade Group >1, which was consistent with other reports (23, 58, 59). A recent meta-analysis of 13 412 radically treated PCa patients suggested that PNI might be an independent prognostic factor which increased the risk of biochemical relapse (60). One study suggested that only sympathetic non-PNI nerve density and non-adrenergic, non-nitrergic PNI nerve density were independently associated with recurrence in PCa (45). PNI creates a specific neoplastic niche, in which tumor progression may be driven by released neurotransmitters and neuropeptides. The analysis of gene expression in cells within the perineural space showed the overexpression of genes involved in anti-apoptotic and proliferation pathways (PIM-2, DAD-1, NFkB) (61). It was found that activated galanin receptors on cancer cells triggered by injured nerves initiated cancer-nerves crosstalk in the head and neck cancer model. The activation of the galanin receptor led to prostaglandin synthesis, facilitated tumor progression and PNI promotion by enhancing neuritogenesis (62). A recent study by Zahalka et al. indicated that adrenergic nerves supported cancer angiogenesis and activated angio-metabolic switch (41). Our study revealed that tumor cell angioinvasion occurred in more advanced cases (pT3 *vs.* pT2, p=0.008), but we did not demonstrate any correlation between angioinvasion and ND. Relatively little is known about molecular mechanisms which drive cancer axonogenesis. The compounds which drive axonogenesis are divided into axon guidance molecules and neurotrophic factors. Single studies concerning various cancers showed that Pro-NGF/NGF (nerve growth factor), BDNF (brain-derived neurotrophic factor), Ephrin B1, neuroligin-3, pleiotrophin, semaphorin 4F were involved in the regulation of tumor innervation (3, 63, 64).

We considered NPY as one of the neurotransmitters possibly involved in PCa neuroendocrine profile and innervation. NPY is the most abundant peptide in the central nervous system, playing numerous peripheral and central functions, e.g. as a sensor and regulator of cell metabolism (65–67). It also promotes the proliferation of the nerve cells of the hippocampus or damaged glial cells (68). The expression of NPY is upregulated in some cancers and supports tumor progression by the activation of various signaling pathways (69). In neuroblastoma and Ewing sarcoma NPY stimulates proliferation and modulates angiogenesis (28, 70). Our study revealed NPY expression in the prostate, PCa, and the neural components of the tumor microenvironment: nerves and autonomic ganglia, similarly to previous research (71–73). Two sources of NPY may be distinguished in cancer cells: paracrine from the nerves within the microenvironment, and autocrine synthesis which allows for neuronal and non-neuronal modulation within tumor components (71). Our study showed that the expression of this peptide was heterogenous within the tumor and higher than in the benign controls. Over 94% of PCa cases showed NPY expression. We analyzed the correlation between NPY and the parameters of tumor innervation. Few clinical studies suggested that NPY innervation was associated with radiation resistance, biochemical recurrence, and PCa-specific death (71). We did not demonstrate the correlation of ND and NPY expression, but NPY immunopositivity was higher in the proximity of PNI, especially in cases with initially high NPY expression. NPY PNI expression was higher than baseline NPY expression in 26.32% of cases. We analyzed all nerve fiber types with a PGP pan-neuronal marker, although selective NPY nerve density in the prostate had been previously reported as higher in high-grade prostate intraepithelial neoplasia than in PCa and in younger men, suggesting that NPY nerves were especially important in prepubertal and pubertal growth (71). We did not observe any correlation between NPY and PCa grade and stage. However, lower NPY had already been found to be associated with less differentiated tumors (74, 75). Additionally, Gleason score of ≤7 and low pro-NPY expression were associated with a lower risk of death than high NPY expression or high Gleason score in one study (76). Importantly, plasma low-molecular-weight proteome profiling showed that NPY and PSA combination revealed the sensitivity of 81.5% and the specificity of 82.2% for PCa diagnosis (77). A recent analysis by Alshalalfa et al. showed the highest risk for uncurable disease in patients with low NPY and ERG-positive subgroup (74). The *TMPRSS2-ERG* fusion is an early and one of the most common genetic abnormalities responsible for tumor invasion and progression affecting growth pathways and regulating differentiation, cell cycle, proliferation, angiogenesis and morphology in PCa (78–80). The overexpression of ERG is a biomarker of fusion, and may be related to unfavorable prognostic factors and decreased survival (80–83). The incidence of fusion significantly differs between the nationalities and our results were similar to other Polish data (26, 84, 85). We performed the immunohistochemical evaluation of ERG status and its associations with clinico-pathological variables and neural parameters finding that ND PGP 9.5 was significantly higher in ERG-positive cancers compared to ERG-negative ones. ND TH did not differ significantly between groups, but PNI was four times more common in ERG+ tumors. A study by Hänze et al. found neural density to be higher in TMPRSS2-ERG-positive cancer and no difference between ND score and PNI (21). The molecular mechanisms of ERG contribution into the neural microenvironment involved regulation compounds which additionally promoted PNI (86). The transcriptional analysis of ERG-positive PCa showed that metabolic changes led to the increased expression of NPY. The role of ERG rearrangement is complex. It supports NPY synthesis in PCa cells but it was also shown to inhibit neuroendocrine differentiation, one of the basic mechanisms driving CRPC (71, 87). *In vitro* studies suggested that it increased glucose uptake in PCa cells which did not express endogenous NPY (65). Our study showed that NPY expression was not correlated with ERG. The population of neuroendocrine cells present in the prostate and PCa contains many peptides, which may be involved in the androgen-dependent and androgen-independent stage of the disease (35). The neuroendocrine differentiation in PCa cells differed between studies and ranged from several to 100% (64). The quantity of chromogranin-positive cells differed between benign prostate and various stages of PCa, being the highest in CRPC. The results of accessing serum or tissue chromogranin levels as a prognostic factor are also contradictory (88–90). Similarly to several previous studies, we used chromogranin to assess neuroendocrine differentiation in PCa. We showed different patterns of chromogranin expression, with high results being obtained in 78.08% of cases based on our criteria. We showed that high neuroendocrine differentiation in PCa occurred 3.84 more often in cases with a high NPY subgroup, which suggests a common neural-neuroendocrine profile and reciprocal regulation within the tumor microenvironment which may influence diagnostics and therapeutic opportunities in the future.

Our study constitutes a complex analysis of several aspects of innervation and neuroendocrine profile in PCa. We assessed the correlations between tumor and the neural microenvironment, evaluating ND and PNI. We showed that the distribution and morphology of tumoral innervation were heterogenous. With the use of our own quantitative method of ND assessment we found that ND was lower in tumoral infiltration than in the exact tumor periphery. Moreover, the density of sympathetic nerve fibers and their proportion to all detected fibers were lower in cancer than in the benign prostate. PNI involves different types and manner of infiltration and occurs more commonly in less differentiated tumors. ERG-positive tumors presented higher innervation, PNI, and tumor stage. NPY signaling seems to be involved in PCa invasion, because of its higher expression in PNI areas. Moreover, tumors with high NPY expression had more abundant neuroendocrine cell populations.

Personalized therapies based on the biological profile create a new direction in PCa treatment (91). Targeting tumor innervation has become a new concept in oncology. Current clinical strategies related to neural impact in cancer involve selective surgical and chemical denervation, systemic therapy, imaging, and pain studies. As regards PCa, some promising data are available concerning β-blockers and the beneficial action of botulinum toxin as treatments downregulating neural signaling (4, 92). Other potentially therapeutic strategies which could affect tumor neurogenesis, axonogenesis, and perineural invasion may be based on targeting soluble neurotrophic factors, axon guidance molecules, and exosomes potentially leading to alteration in tumor growth, metastases, cancer-induced pain, angiogenesis, and nerve-immune crosstalk. Currently, the NGF/Trk signaling pathway is the most studied and most promising, since preclinical studies showed that anti-NGF antibody inhibited the growth and metastases of PCa (93). An interesting treatment option would also involve NGF gene silencing with gold nanocluster-assisted delivery of siRNA, and anti-NGF antibody- based therapies (94). It was also shown that the other neurotrophic factor, GDNF released by prostate cancer fibroblasts was involved in docetaxel tumor chemoresistance (95). The new direction in cancer-nerve crosstalk may be either targeting the perineural niche in cancer, which contributes to oncogenic communication between cancer and the neuro-immune system. The disruption of the mutual regulation between neural microenvironment (axonogenesis, neurogenesis) and leukocytes (immune evasion) is an interesting topic for further investigation in the context of immunotherapy, which significantly increases the effectiveness of oncological treatment (3). Research concerning the mechanism of cancer innervation may also broaden the knowledge about the treatment and prevention of neurological side effects caused by systemic therapy (96). It is crucial to identify neuro-molecular pathways involved in neural-cancer crosstalk and interactions to generate new therapeutic targets (42). Further research on axonogenesis should be performed in large cohorts of patients, especially in the context of clinico-pathological data and clinical relevance.

## Data Availability Statement

The original contributions presented in the study are included in the article/supplementary material. Further inquiries can be directed to the corresponding author.

## Ethics Statement

The studies involving human participants were reviewed and approved by Medical University of Gdańsk Bioethics Committee (NKBBN/448/2015). The ethics committee waived the requirement of written informed consent for participation.

## Author Contributions

Design and conception: DS and EI-S. Data collection and analyses: DS, AS, JG, WR, EI-S. Writing of manuscript: DS and EI-S. Editing and reviewing the manuscript: DS, JG, AS, WR, and EI-S. Final approval of the version to be published: DS, JG, AS, WR, and EI-S. All authors contributed to the article and approved the submitted version.

## Funding

This research was supported by the Medical University of Gdansk (internal grant 02-0562-07/324) and University of Warmia and Mazury in Olsztyn.

## Conflict of Interest

The authors declare that the research was conducted in the absence of any commercial or financial relationships that could be construed as a potential conflict of interest.

## References

[1] Mauffrey P Tchitchek N Barroca V Bemelmans A Firlej V Allory Y. Progenitors From the Central Nervous System Drive Neurogenesis in Cancer. Nature (2019) 569:672–8. 10.1038/s41586-019-1219-y 31092925

[2] Magnon C Hall SJ Lin J Xue X Gerber L Freedland SJ. Autonomic Nerve Development Contributes to Prostate Cancer Progression. Science (2013) 341:1236361. 10.1126/science.1236361 23846904

[3] Cervantes-Villagrana RD Albores-García D Cervantes-Villagrana AR García-Acevez SJ. Tumor-Induced Neurogenesis and Immune Evasion as Targets of Innovative Anti-Cancer Therapies. Signal Transduct Target Ther (2020) 5:1–23. 10.1038/s41392-020-0205-z 32555170 PMC7303203

[4] Coarfa C Florentin D Putluri N Ding Y Au J He D. Influence of the Neural Microenvironment on Prostate Cancer. Prostate (2018) 78:128–39. 10.1002/pros.23454 PMC5836952 29131367

[5] Sejda A Sigorski D Gulczyński J Wesołowski W Kitlińska J Iżycka-Świeszewska E. Complexity of Neural Component of Tumor Microenvironment in Prostate Cancer. Pathobiology (2020) 87:87–99. 10.1159/000505437 32045912

[6] Ayala GE Wheeler TM Shine HD Schmelz M Frolov A Chakraborty S. In Vitro Dorsal Root Ganglia and Human Prostate Cell Line Interaction: Redefining Perineural Invasion in Prostate Cancer. Prostate (2001) 49:213–23. 10.1002/pros.1137 11746267

[7] Frisbie JH Binard J. Low Prevalence of Prostatic Cancer Among Myelopathy Patients. J Am Paraplegia Soc (1994) 17:148–9. 10.1080/01952307.1994.11735926 7964711

[8] Shim HB Jung TY Lee JK Ku JH. Prostate Activity and Prostate Cancer in Spinal Cord Injury. Prostate Cancer Prostatic Dis (2006) 9:115–20. 10.1038/sj.pcan.4500865 16534510

[9] Ayala GE Dai H Powell M Li R Ding Y Wheeler TM. Cancer-Related Axonogenesis and Neurogenesis in Prostate Cancer. Clin Cancer Res (2008) 14:7593–603. 10.1158/1078-0432.CCR-08-1164 19047084

[10] Tavukçu HH Aytac O Atug F. Nerve-Sparing Techniques and Results in Robot-Assisted Radical Prostatectomy. Investig Clin Urol (2016) 57:S172–84. 10.4111/icu.2016.57.S2.S172 PMC5161020 27995221

[11] Ciftci S Yilmaz H Ciftci E Simsek E Ustuner M Yavuz U. Perineural Invasion in Prostate Biopsy Specimens Is Associated With Increased Bone Metastasis in Prostate Cancer. Prostate (2015) 75:1783–9. 10.1002/pros.23067 26286637

[12] Brown IS. Pathology of Perineural Spread. J Neurol Surg B Skull Base (2016) 77:124–30. 10.1055/s-0036-1571837 PMC4846404 27123388

[13] Chen S-H Zhang B-Y Zhou B Zhu C-Z Sun L-Q Feng Y-J. Perineural Invasion of Cancer: A Complex Crosstalk Between Cells and Molecules in the Perineural Niche. Am J Cancer Res (2019) 9:1–21. 30755808 PMC6356921

[14] Mancino M Ametller E Gascón P Almendro V. The Neuronal Influence on Tumor Progression. Biochim Biophys Acta - Rev Cancer (2011) 1816:105–18. 10.1016/J.BBCAN.2011.04.005 21616127

[15] Bakst RL Wong RJ. Mechanisms of Perineural Invasion. J Neurol Surg B Skull Base (2016) 77:96–106. 10.1055/s-0036-1571835 27123385 PMC4846411

[16] Boilly B Faulkner S Jobling P Hondermarck H. Nerve Dependence: From Regeneration to Cancer. Cancer Cell (2017) 31:342–54. 10.1016/j.ccell.2017.02.005 28292437

[17] Albo D Akay CL Marshall CL Wilks JA Verstovsek G Liu H. Neurogenesis in Colorectal Cancer Is a Marker of Aggressive Tumor Behavior and Poor Outcomes. Cancer (2011) 117:4834–45. 10.1002/cncr.26117 21480205

[18] Olar A He D Florentin D Ding Y Wheeler T Ayala G. Biological Correlates of Prostate Cancer Perineural Invasion Diameter. Hum Pathol (2014) 45:1365–9. 10.1016/j.humpath.2014.02.011 PMC4492300 24768607

[19] Nielsen MM Tolbod LP Borre M Høyer S Harms HJ Sørensen J. The Relationship Between Tumor Aggressiveness and Cholinergic PET Imaging in Prostate Cancer Tissue. A Proof-of-Concept Study. Am J Nucl Med Mol Imaging (2019) 9:185–92. PMC6627478 31328024

[20] Iwasaki T Hiraoka N Ino Y Nakajima K Kishi Y Nara S. Reduction of Intrapancreatic Neural Density in Cancer Tissue Predicts Poorer Outcome in Pancreatic Ductal Carcinoma. Cancer Sci (2019) 110:1491–502. 10.1111/cas.13975 PMC6447831 30776178

[21] Hänze J Rexin P Jakubowski P Schreiber H Heers H Lingelbach S. Prostate Cancer Tissues With Positive TMPRSS2-ERG-Gene-Fusion Status may Display Enhanced Nerve Density. Urol Oncol Semin Orig Investig (2018) 38(1):3.e7–3.e15. 10.1016/j.urolonc.2018.07.019 30241953

[22] Pundavela J Roselli S Faulkner S Attia J Scott RJ Thorne RF. Nerve Fibers Infiltrate the Tumor Microenvironment and Are Associated With Nerve Growth Factor Production and Lymph Node Invasion in Breast Cancer. Mol Oncol (2015) 9:1626–35. 10.1016/J.MOLONC.2015.05.001 PMC5528785 26009480

[23] Reeves F Hovens CM Harewood L Battye S Peters JS Costello AJ. Does Perineural Invasion in a Radical Prostatectomy Specimen Predict Biochemical Recurrence in Men With Prostate Cancer? Can Urol Assoc J (2015) 9:E252–5. 10.5489/cuaj.2619 PMC4439219 26029290

[24] Sizemore GM Pitarresi JR Balakrishnan S Ostrowski MC. The ETS Family of Oncogenic Transcription Factors in Solid Tumours. Nat Rev Cancer (2017) 17:337–51. 10.1038/nrc.2017.20 28450705

[25] Kristensen G Røder MA Berg KD Elversang J Iglesias-Gato D Moreira J. Predictive Value of Combined Analysis of Pro-NPY and ERG in Localized Prostate Cancer. APMIS (2018) 126:804–13. 10.1111/apm.12886 30191621

[26] Adamo P Ladomery MR. The Oncogene ERG: A Key Factor in Prostate Cancer. Oncogene (2016) 35:403–14. 10.1038/onc.2015.109 25915839

[27] Scaravilli M Koivukoski S Latonen L. Androgen-Driven Fusion Genes and Chimeric Transcripts in Prostate Cancer. Front Cell Dev Biol (2021) 9:1–14, 623809. 10.3389/fcell.2021.623809 PMC7900491 33634124

[28] Kitlinska J Abe K Kuo L Pons J Yu M Li L. Differential Effects of Neuropeptide Y on the Growth and Vascularization of Neural Crest-Derived Tumors. Cancer Res (2005) 65:1719–28. 10.1158/0008-5472.CAN-04-2192 15753367

[29] Farach A Ding Y Lee M Creighton C Delk NA Ittmann M. Neuronal Trans-Differentiation in Prostate Cancer Cells. Prostate (2016) 76:1312–25. 10.1002/pros.23221 PMC5815867 27403603

[30] Li Q Zhang CS Zhang Y. Molecular Aspects of Prostate Cancer With Neuroendocrine Differentiation. Chin J Cancer Res (2016) 28:122–9. 10.3978/j.issn.1000-9604.2016.01.02 PMC4779763 27041934

[31] Humphrey PA Moch H Cubilla AL Ulbright TM Reuter VE. The 2016 WHO Classification of Tumours of the Urinary System and Male Genital Organs—Part B: Prostate and Bladder Tumours. Eur Urol (2016) 70:106–19. 10.1016/j.eururo.2016.02.028 26996659

[32] Cassoni AM. TNM Classification of Malignant Tumours. Clin Oncol (1998) 10:61. 10.1016/s0936-6555(98)80120-9

[33] Current CAP Guidelines | College of American Pathologists. Available at: https://www.cap.org/protocols-and-guidelines/current-cap-guidelines (Accessed March 10, 2021).

[34] Pirker R Pereira JR von Pawel J Krzakowski M Ramlau R Park K. EGFR Expression as a Predictor of Survival for First-Line Chemotherapy Plus Cetuximab in Patients With Advanced Non-Small-Cell Lung Cancer: Analysis of Data From the Phase 3 FLEX Study. Lancet Oncol (2012) 13:33–42. 10.1016/S1470-2045(11)70318-7 22056021

[35] Ishida E Nakamura M Shimada K Tasaki M Konishi N. Immunohistochemical Analysis of Neuroendocrine Differentiation in Prostate Cancer. Pathobiology (2009) 76:30–8. 10.1159/000178153 19188748

[36] Barbonetti A D’Andrea S Martorella A Felzani G Francavilla S Francavilla F. Risk of Prostate Cancer in Men With Spinal Cord Injury: A Systematic Review and Meta-Analysis. Asian J Androl (2018) 20:555–60. 10.4103/aja.aja_31_18 PMC6219305 29956686

[37] Zahalka AH Frenette PS. Nerves in Cancer. Nat Rev Cancer (2020) 20:143–57. 10.1038/s41568-019-0237-2 PMC7709871 31974491

[38] Zhang D Park D Zhong Y Lu Y Rycaj K Gong S. Stem Cell and Neurogenic Gene-Expression Profiles Link Prostate Basal Cells to Aggressive Prostate Cancer. Nat Commun (2016) 7:10798. 10.1038/ncomms10798 26924072 PMC4773505

[39] Bautista M Krishnan A. The Autonomic Regulation of Tumor Growth and the Missing Links. Front Oncol (2020) 10:744. 10.3389/fonc.2020.00744 32477953 PMC7237572

[40] Reavis HD Chen HI Drapkin R. Tumor Innervation: Cancer Has Some Nerve. Trends Cancer (2020) 0:1059–67. 10.1016/j.trecan.2020.07.005 PMC7688507 32807693

[41] Zahalka AH Arnal-Estapé A Maryanovich M Nakahara F Cruz CD Finley LWS. Adrenergic Nerves Activate an Angio-Metabolic Switch in Prostate Cancer. Science (2017) 358:321–6. 10.1126/science.aah5072 PMC5783182 29051371

[42] Demir IE Reyes CM Alrawashdeh W Ceyhan GO Deborde S Friess H. Clinically Actionable Strategies for Studying Neural Influences in Cancer. Cancer Cell (2020) 38:11–4. 10.1016/j.ccell.2020.05.023 32531270

[43] Gillespie S Monje M. The Neural Regulation of Cancer. Annu Rev Cancer Biol (2020) 4:371–90. 10.1146/annurev-cancerbio-030419-033349

[44] Fernández EV Price DK Figg WD. Prostate Cancer Progression Attributed to Autonomic Nerve Development. Cancer Biol Ther (2013) 14:1005–6. 10.4161/cbt.26339 PMC3925654 24025357

[45] Reeves FA Battye S Roth H Peters JS Hovens C Costello AJ. Prostatic Nerve Subtypes Independently Predict Biochemical Recurrence in Prostate Cancer. J Clin Neurosci (2019) 63:213–9. 10.1016/j.jocn.2019.01.052 30772200

[46] Lu R Fan C Shangguan W Liu Y Li Y Shang Y. Neurons Generated From Carcinoma Stem Cells Support Cancer Progression. Signal Transduct Target Ther (2017) 2:1–10. 10.1038/sigtrans.2016.36 PMC5657421 29263908

[47] He D Manzoni A Florentin D Fisher W Ding Y Lee MJ. Biologic Effect of Neurogenesis in Pancreatic Cancer. Hum Pathol (2016) 52:182–9. 10.1016/j.humpath.2016.02.001 26980040

[48] Rowe CW Dill T Griffin N Jobling P Faulkner S Paul JW. Innervation of Papillary Thyroid Cancer and its Association With Extra-Thyroidal Invasion. Sci Rep (2020) 10:1–9. 10.1038/s41598-020-58425-5 32001748 PMC6992619

[49] You H Shang W Min X Weinreb J Li Q Leapman M. Sight and Switch Off: Nerve Density Visualization for Interventions Targeting Nerves in Prostate Cancer. Sci Adv (2020) 6:eaax6040. 10.1126/sciadv.aax6040 32076639 PMC7002130

[50] Zhang L Wu LL Huan HB Chen XJ Wen XD Yang DP. Sympathetic and Parasympathetic Innervation in Hepatocellular Carcinoma. Neoplasma (2017) 64:840–6. 10.4149/neo_2017_605 28895408

[51] Cooperberg MR Pasta DJ Elkin EP Litwin MS Latini DM Duchane J. The University of California, San Francisco Cancer of the Prostate Risk Assessment Score: A Straightforward and Reliable Preoperative Predictor of Disease Recurrence After Radical Prostatectomy. J Urol (2005) 173:1938–42. 10.1097/01.ju.0000158155.33890.e7 PMC2948569 15879786

[52] D’Amico AV Whittington R Bruce Malkowicz S Schultz D Blank K Broderick GA. Biochemical Outcome After Radical Prostatectomy, External Beam Radiation Therapy, or Interstitial Radiation Therapy for Clinically Localized Prostate Cancer. J Am Med Assoc (1998) 280:969–74. 10.1001/jama.280.11.969 9749478

[53] Niklas C Saar M Berg B Steiner K Janssen M Siemer S. Da Vinci and Open Radical Prostatectomy: Comparison of Clinical Outcomes and Analysis of Insurance Costs. Urol Int (2016) 96:287–94. 10.1159/000431104 26159050

[54] Patel VR Shah S Arend D. Histopathologic Outcomes of Robotic Radical Prostatectomy. ScientificWorldJournal (2006) 6:2566–72. 10.1100/tsw.2006.397 PMC5917347 17619732

[55] Strom P Nordström T Delahunt B Samaratunga H Gronberg H Egevad L. Prognostic Value of Perineural Invasion in Prostate Needle Biopsies: A Population-Based Study of Patients Treated by Radical Prostatectomy. J Clin Pathol (2020) 73:630–5. 10.1136/jclinpath-2019-206300 PMC7513266 32034057

[56] Wu S Lin X Lin SX Lu M Deng T Wang Z. Impact of Biopsy Perineural Invasion on the Outcomes of Patients Who Underwent Radical Prostatectomy: A Systematic Review and Meta-Analysis. Scand J Urol (2019) 53:287–94. 10.1080/21681805.2019.1643913 31401922

[57] Kraus RD Barsky A Ji L Garcia Santos PM Cheng N Groshen S. The Perineural Invasion Paradox: Is Perineural Invasion an Independent Prognostic Indicator of Biochemical Recurrence Risk in Patients With Pt2n0r0 Prostate Cancer? A Multi-Institutional Study. Adv Radiat Oncol (2019) 4:96–102. 10.1016/j.adro.2018.09.006 30706016 PMC6349660

[58] Lee IH Roberts R Shah RB Wojno KJ Wei JT Sandler HM. Perineural Invasion is a Marker for Pathologically Advanced Disease in Localized Prostate Cancer. Int J Radiat Oncol Biol Phys (2007) 68:1059–64. 10.1016/j.ijrobp.2007.01.039 PMC2771329 17398032

[59] Zareba P Flavin R Isikbay M Rider JR Gerke TA Finn S. Perineural Invasion and Risk of Lethal Prostate Cancer. Cancer Epidemiol Biomarkers Prev (2017) 26:719–26. 10.1158/1055-9965.EPI-16-0237 PMC5413395 28062398

[60] Zhang LJ Wu B Zha ZL Qu W Zhao H Yuan J. Perineural Invasion as an Independent Predictor of Biochemical Recurrence in Prostate Cancer Following Radical Prostatectomy or Radiotherapy: A Systematic Review and Meta-Analysis. BMC Urol (2018) 18:1–10. 10.1186/s12894-018-0319-6 29390991 PMC5796578

[61] Ayala GE Dai H Ittmann M Li R Powell M Frolov A. Growth and Survival Mechanisms Associated With Perineural Invasion in Prostate Cancer. Cancer Res (2004) 64:6082–90. 10.1158/0008-5472.CAN-04-0838 15342391

[62] Scanlon CS Banerjee R Inglehart RC Liu M Russo N Hariharan A. Galanin Modulates the Neural Niche to Favour Perineural Invasion in Head and Neck Cancer. Nat Commun (2015) 6:6885. 10.1038/ncomms7885 25917569 PMC4476386

[63] Kerekes N Landry M Lundmark K Hökfelt T. Effect of NGF, BDNF, bFGF, aFGF and Cell Density on NPY Expression in Cultured Rat Dorsal Root Ganglion Neurones. J Auton Nerv Syst (2000) 81:128–38. 10.1016/S0165-1838(00)00115-6 10869711

[64] Liang Y Wang W Huang J Tan H Liu T Shang C. Potential Role of Semaphorin 3A and Its Receptors in Regulating Aberrant Sympathetic Innervation in Peritoneal and Deep Infiltrating Endometriosis. PLoS One (2015) 10:e0146027. 10.1371/journal.pone.0146027 26720585 PMC4697795

[65] Massoner P Kugler KG Unterberger K Kuner R Mueller LAJ Fälth M. Characterization of Transcriptional Changes in ERG Rearrangement-Positive Prostate Cancer Identifies the Regulation of Metabolic Sensors Such as Neuropeptide Y. PLoS One (2013) 8:e55207. 10.1371/journal.pone.0055207 23390522 PMC3563644

[66] Hirsch D Zukowska Z. NPY and Stress 30 Years Later: The Peripheral View. Cell Mol Neurobiol (2012) 32:645–59. 10.1007/s10571-011-9793-z PMC3492947 22271177

[67] Zhang L Bijker MS Herzog H. The Neuropeptide Y System: Pathophysiological and Therapeutic Implications in Obesity and Cancer. Pharmacol Ther (2011) 131:91–113. 10.1016/j.pharmthera.2011.03.011 21439311

[68] Decressac M Wright B David B Tyers P Jaber M Barker RA. Exogenous Neuropeptide Y Promotes *In Vivo* Hippocampal Neurogenesis. Hippocampus (2011) 21:233–8. 10.1002/hipo.20765 20095007

[69] Körner M Reubi JC. NPY Receptors in Human Cancer: A Review of Current Knowledge. Peptides (2007) 28:419–25. 10.1016/j.peptides.2006.08.037 17223228

[70] Czarnecka M Trinh E Lu C Kuan-Celarier A Galli S Hong S-H. Neuropeptide Y Receptor Y5 as an Inducible Pro-Survival Factor in Neuroblastoma: Implications for Tumor Chemoresistance. Oncogene (2015) 34:3131–43. 10.1038/onc.2014.253 PMC4333135 25132261

[71] Ding Y Lee M Gao Y Bu P Coarfa C Miles B. Neuropeptide Y Nerve Paracrine Regulation of Prostate Cancer Oncogenesis and Therapy Resistance. Prostate (2020) pros.24081:58–71. 10.1002/pros.24081 PMC7756863 33022812

[72] Martin R Fraile B Peinado F Arenas MI Elices M Alonso L. Immunohistochemical Localization of Protein Gene Product 9.5, Ubiquitin and Neuropeptide Y Immunoreactivities in Epithelial and Neuroendocrine Cells From Normal and Hyperplastic Human Prostate. J Histochem Cytochem (2000) 48:1121–30. 10.1177/002215540004800809 10898805

[73] Rasiah KK Kench JG Gardiner-Garden M Biankin AV Golovsky D Brenner PC. Aberrant Neuropeptide Y and Macrophage Inhibitory Cytokine-1 Expression are Early Events in Prostate Cancer Development and are Associated With Poor Prognosis. Cancer Epidemiol Biomarkers Prev (2006) 15:711–6. 10.1158/1055-9965.EPI-05-0752 16614113

[74] Alshalalfa M Nguyen PL Beltran H Chen WS Davicioni E Zhao SG. Transcriptomic and Clinical Characterization of Neuropeptide Y Expression in Localized and Metastatic Prostate Cancer: Identification of Novel Prostate Cancer Subtype With Clinical Implications. Eur Urol Oncol (2019) 2:405–12. 10.1016/j.euo.2019.05.001 PMC7597937 31164324

[75] Liu J Kahri AI Heikkilä P Voutilainen R. Regulation of Neuropeptide Y mRNA Expression in Cultured Human Pheochromocytoma Cells. Eur J Endocrinol (1999) 141:431–5. 10.1530/eje.0.1410431 10526260

[76] Iglesias-Gato D Wikström P Tyanova S Lavallee C Thysell E Carlsson J. The Proteome of Primary Prostate Cancer. Eur Urol (2016) 69:942–52. 10.1016/j.eururo.2015.10.053 26651926

[77] Ueda K Tatsuguchi A Saichi N Toyama A Tamura K Furihata M. Plasma Low-Molecular-Weight Proteome Profiling Identified Neuropeptide-Y as a Prostate Cancer Biomarker Polypeptide. J Proteome Res (2013) 12:4497–506. 10.1021/pr400547s 23991666

[78] Perner S Mosquera JM Demichelis F Hofer MD Paris PL Simko J. TMPRSS2-ERG Fusion Prostate Cancer: An Early Molecular Event Associated With Invasion. Am J Surg Pathol (2007) 31:882–8. 10.1097/01.pas.0000213424.38503.aa 17527075

[79] Okoń K Dyduch G Białas MB Milian-Ciesielska K Szpor J Leszczyńska I. Image Analysis Discloses Differences in Nuclear Parameters Between ERG+ and ERG– Prostatic Carcinomas. Polish J Pathol (2020) 71:20–9. 10.5114/pjp.2020.95412 32429651

[80] Wang Z Wang Y Zhang J Hu Q Zhi F Zhang S. Significance of the TMPRSS2:ERG Gene Fusion in Prostate Cancer. Mol Med Rep (2017) 16:5450–8. 10.3892/mmr.2017.7281 PMC5647090 28849022

[81] Minner S Enodien M Sirma H Luebke AM Krohn A Mayer PS. ERG Status Is Unrelated to PSA Recurrence in Radically Operated Prostate Cancer in the Absence of Antihormonal Therapy. Clin Cancer Res (2011) 17:5878–88. 10.1158/1078-0432.CCR-11-1251 21791629

[82] Küronya Z Sükösd F Varga L Bíró K Gyergyay F Géczi L. ERG Expression can Predict the Outcome of Docetaxel Combinedwith Androgen Deprivation Therapy in Metastatic Hormone-Sensitiveprostate Cancer. Urol Oncol Semin Orig Investig (2019) 37:289.e1–9. 10.1016/j.urolonc.2018.12.007 30679082

[83] Rezk M Chandra A Addis D Møller H Youssef M Dasgupta P. *ETS-Related Gene* (*ERG*) Expression as a Predictor of Oncological Outcomes in Patients With High-Grade Prostate Cancer Treated With Primary Androgen Deprivation Therapy: A Cohort Study. BMJ Open (2019) 9:e025161. 10.1136/bmjopen-2018-025161 PMC6429920 30852544

[84] Kaczmarczyk K Dyduch G Białas M Demczuk S Szopiński T Chłosta P. Frequency of ERG-Positive Prostate Carcinoma in Poland. Polish J Pathol (2013) 3:175–9. 10.5114/pjp.2013.38134 24166602

[85] Abdel-Hady A El-Hindawi A Hammam O Khalil H Diab S El-Aziz SA. Expression of ERG Protein and TMRPSS2-ERG Fusion in Prostatic Carcinoma in Egyptian Patients. Open Access Maced J Med Sci (2017) 5:147–54. 10.3889/oamjms.2017.037 PMC5420765 28507619

[86] Tian TV Tomavo N Huot L Flourens A Bonnelye E Flajollet S. Identification of Novel TMPRSS2:ERG Mechanisms in Prostate Cancer Metastasis: Involvement of MMP9 and PLXNA2. Oncogene (2014) 33:2204–14. 10.1038/onc.2013.176 23708657

[87] Mounir Z Lin F Lin VG Korn JM Yu Y Valdez R. TMPRSS2:ERG Blocks Neuroendocrine and Luminal Cell Differentiation to Maintain Prostate Cancer Proliferation. Oncogene (2015) 34:3815–25. 10.1038/onc.2014.308 25263440

[88] Berruti A Mosca A Porpiglia F Bollito E Tucci M Vana F. Chromogranin A Expression in Patients With Hormone Naïve Prostate Cancer Predicts the Development of Hormone Refractory Disease. J Urol (2007) 178:838–43. 10.1016/j.juro.2007.05.018 17631319

[89] Cindolo L Cantile M Franco R Chiodini P Schiavo G Forte I. Parallel Determination of Neurod1, Chromogranin-A, KI67 and Androgen Receptor Expression in Surgically Treated Prostate Cancers. Int Braz J Urol (2011) 37:57–66. 10.1590/S1677-55382011000100008 21385481

[90] Guo Z Wang Y Xiang S Wang S Chan FL. Chromogranin A is a Predictor of Prognosis in Patients With Prostate Cancer: A Systematic Review and Meta-Analysis. Cancer Manag Res (2019) 11:2747–58. 10.2147/CMAR.S190678 PMC6497897 31114331

[91] Sigorski D Iżycka-Świeszewska E Bodnar L. Poly(ADP-Ribose) Polymerase Inhibitors in Prostate Cancer: Molecular Mechanisms, and Preclinical and Clinical Data. Target Oncol (2020) 15(6): 709–22. 10.1007/s11523-020-00756-4 33044685 PMC7701127

[92] Lu H Liu X Guo F Tan S Wang G Liu H. Impact of Beta-Blockers on Prostate Cancer Mortality: A Meta-Analysis of 16,825 Patients. Onco Targets Ther (2015) 8:985–90. 10.2147/OTT.S78836 PMC4425323 25995645

[93] Warrington RJ Lewis KE. Natural Antibodies Against Nerve Growth Factor Inhibit *In Vitro* Prostate Cancer Cell Metastasis. Cancer Immunol Immunother (2011) 60:187–95. 10.1007/s00262-010-0934-x PMC11028632 20976447

[94] Lei Y Tang L Xie Y Xianyu Y Zhang L Wang P. Gold Nanoclusters-Assisted Delivery of NGF siRNA for Effective Treatment of Pancreatic Cancer. Nat Commun (2017) 8:1–15. 10.1038/ncomms15130 28440296 PMC5414062

[95] Huber RM Lucas JM Gomez-Sarosi LA Coleman I Zhao S Coleman R. DNA Damage Induces GDNF Secretion in the Tumor Microenvironment With Paracrine Effects Promoting Prostate Cancer Treatment Resistance. Oncotarget (2015) 6:2134–47. 10.18632/oncotarget.3040 PMC4385841 25575823

[96] Monje M Borniger JC D’Silva NJ Deneen B Dirks PB Fattahi F. Roadmap for the Emerging Field of Cancer Neuroscience. Cell (2020) 181:219–22. 10.1016/j.cell.2020.03.034 PMC7286095 32302564

